# Early detection of dark-affected plant mechanical responses using enhanced electrical signals

**DOI:** 10.1186/s13007-024-01169-4

**Published:** 2024-03-26

**Authors:** Hongping Li, Nikou Fotouhi, Fan Liu, Hongchao Ji, Qian Wu

**Affiliations:** 1 College of Computer Science and Technology (College of Data Science), Taiyuan University of Technology, Jinzhong, 030600 Shanxi China; 2 Shenzhen Branch, Guangdong Laboratory of Lingnan Modern Agriculture, Key Laboratory of Synthetic Biology, Ministry of Agriculture and Rural Affairs, Agricultural Genomics Institute at Shenzhen, Chinese Academy of Agricultural Sciences, Shenzhen, 518120 Guangdong China; 3 Desai Sethi Urology Institute, Sylvester Comprehensive Cancer Center, University of Miami Miller School of Medicine, Miami, FL 33136 USA

**Keywords:** Environmental stress, Plant electrical signal, Machine learning, Classification, Data augmentation

## Abstract

**Background:**

Mechanical damage to plants triggers local and systemic electrical signals that are eventually decoded into plant defense responses. These responses are constantly affected by other environmental stimuli in nature, for instance, light fluctuation. In recent years, studies on decoding plant electrical signals powered by various machine learning models are increasing in a sense of early prediction or detection of different environmental stresses that threaten plant growth or crop yields. However, the main bottleneck is the low-throughput nature of plant electrical signals, making it challenging to obtain a substantial amount of training data. Consequently, training these models with small datasets often leads to unsatisfactory performance.

**Results:**

In the present work, we set out to decode wound-induced electrical signals (also termed slow wave potentials, SWPs) from plants that are deprived of light to different extents. Using non-invasive electrophysiology, we separately collected sets of local and distal SWPs from the treated plants. Then, we proposed a workflow based on few-shot learning to automatically identify SWPs. This workflow incorporates data preprocessing, feature extraction, data augmentation and classifier training. We established the integral and the first-order derivative as features for efficiently classifying SWPs. We then proposed an Adversarial Autoencoder (AAE) structure to augment the SWP samples. Combining them, the Random Forest classifier allowed remarkable classification accuracies of 0.99 for both local and systemic SWPs. In addition, in comparison to two other reported methods, our proposed AAE structure enabled better classification results using our tested features and classifiers.

**Conclusions:**

The results of this study establish new features for efficiently classifying wound-induced electrical signals, which allow for distinguishing dark-affected local and systemic plant wound responses. We also propose a new data augmentation structure to generate virtual plant electrical signals. The methods proposed in this study could be further applied to build models for crop plants using electrical signals as inputs, and also to process other small-scale signals.

**Supplementary Information:**

The online version contains supplementary material available at 10.1186/s13007-024-01169-4.

## Background

In nature, plants are constantly challenged by various environmental stimuli. Sub-optimal conditions like drought, salinity, rainfall, wind, light irradiation, as well as attacks by pathogens or insects cause severe damage to crop plants all over the world every year, which eventually leads to a great threat to food security [1, 2]. To deal with stressing factors, plants have evolved sophisticated strategies to defend themselves, in the meantime, maintaining their overall fitness under unfavored circumstances. Out of all these environmental stimuli, mechanical damages elicited by wounding of chewing insects not only break down tissue integrity and dampen crop growth, but also facilitate pathogen infections and greatly reduce the yield [3]. Therefore, an increasing number of studies were carried out to decipher how plants coordinate themselves in response to mechanical damages within the spot of injury as well as throughout the bodies, aiming at designing stress resilient plants.

As early as last decades, it was already observed that damages to plants triggered electrical signals that propagated to a distance away from the wounds [4]. These kinds of signals exist widespread across plant kingdoms, for instance, in the wounded tomato plants [5], the excised cucumber hypocotyls [6] and the crushed or chewed *Arabidopsis* leaves [7]. Interestingly, in these cases, the electrical signals typified the signature of the variation potentials (VPs), which has a steep depolarization phase and a slow repolarization phase. Besides VPs, damage also triggers action potentials (APs) and systemic potentials (SPs) in various plant species. For example, APs and SPs were detected on the stems and leaves of *Vicia faba* and *Hordeum vulgare*, respectively, upon herbivory [8]. However, the biological relevance of the electrical signals was largely unknown until the characterization of clade three *Arabidopsis* glutamate receptor-like (*GLRs*) genes [7]. Upon leaf wounding, *Arabidopsis* plants with impaired function of *GLR3.3*, *GLR3.6* and *GLR3.1* genes failed to propagate electrical signals to the distal intact leaves. Accordingly, defense responses mediated by the defense hormone jasmonate were largely reduced [7].

Wound-induced electrical signals are also termed as slow wave potentials (SWPs), in light of the different features in comparison with other electrical reactions. With the identification of more components in wound-induced electrical signaling, SWPs were further characterized and decoded using multiple parameters. Duration of SWPs was established as a major parameter in indicating the levels of defense activation in a couple of loss-of-function mutants. Unlike *glr* mutants, in *Arabidopsis H*^+^*-ATPase AHA1* mutants, the longer duration of SWPs corresponded to a stronger defense response [9]. In another *Arabidopsis* mutants defective in the mechanosensitive channels (MSLs), besides the SWP duration, SWP repolarization maxima was another key feature corresponded to the defense activation [10]. Interestingly, in the rice *glr3.4* loss-of-function mutants, peak amplitude of the SWP determined the defense levels in response to wounding [11]. Moreover, in two *irregular xylem* (*irx*) mutants *irx3* and *irx5* showing altered systemic defense responses, wound-induced SWPs displayed several different characteristics compared to wild-type plants, including the detected depolarization spikes prior to the main SWP signal, the slope of the principal depolarization and also the velocity of SWPs [12]. Therefore, the features of SWPs are tightly correlated with the defense activities in plants and are potentialized in indicating how plants react to wound stimuli under different circumstances. However, little information is available in the direction of decoding SWP features, which not only limits our understanding on how plants utilize SWPs to initiate systemic defense responses, but also restricts the potential application of SWPs in indicating the variable mechanical responses of plants when they are challenged by biotic or abiotic stresses.

In recent years, plant electrical signals are emerging as stimuli-specific phenotypes to differentiate plants from a wide range of environmental stimuli [13]. Moreover, as electrical signals are often produced rapidly upon stimuli, detection and classification of plant electrical signals also allow early diagnosis of the health status of plants before visible symptoms [14, 15]. In particular, machine learning-coupled strategies showed great power in analyzing and classifying plant electrical signals under various conditions. For example, Chatterjee et al. [16] extracted 11 statistical features from the electrical signals of tomato plants under different stimuli (NaCl, H_2_SO_4_ and O_3_) and then classified them using several machine learning algorithms, i.e. Fisher’s linear discriminant analysis (LDA), quadratic discriminant analysis (QDA), naive Bayes classifier, and Mahalonobis classifier. The best accuracy of 73.67% was archived for the multiple classifications. More recently, an improved accuracy of above 90% was reached when four different curve fitting methods (Polynomial, Gaussian, Fourier and Exponential) were used to obtain the coefficients of the fitted model as features in combination with LDA and QDA as the classifiers [17]. In addition, Qin et al. [18] proposed a CNN structure to extract features and classify electrical signals of wheat plants under salt stress, reaching a classification accuracy of 92.3%. Following this, they further introduced a 1D-CNN-LSTM model to classify the electrical signals of wheat plants under serial concentrations of NaCl [19]. In another study, an interval algorithm was used to reduce the dimension of the input electrical signal in combination with the SVM classifier [20]. More recently, to classify various nutrient deficiencies in tomato plants, Intrinsic Mode Functions (IMFs) of electrical signals from tomato plants cultured under various nutrient deficiencies (Ca, Fe, Mn and N) were extracted using Empirical Mode Decomposition (EMD). An average accuracy rate of 98.5% was reached using descriptive statistics and the bi-level measurements of IMF groups as features [21]. Despite the growing number of studies on decoding plant electrical signals, the successful utilization of plant electrical signals to predict how plants react to various stress conditions is still constrained by the low throughput of signals that can be used to generate reliable models.

In this study, we sought to determine if wound-induced local and systemic SWPs could be classified when plants are deprived of light to different extents. On the one hand, light qualities are crucial in determining plant growth [22]. On the other hand, geographical differences at different altitudes render plants with variable access to sunlight. Light fluctuation also occurs when smaller plants are shaded by the taller ones grown under high densities [23]. Whether plants grown under less sunlight react differently to mechanical stress compared to those receiving more sunlight remains uncharacterized. To address these questions, we simulated the variable light access in nature by treating the *Arabidopsis* plants under dark conditions to different extents, took advantage of the non-invasive electrophysiology to measure SWPs in the wounded and the distal connected leaves [24], extracted features from the traces and finally classified them using improved machine learning models. Notably, we also proposed a new network structure to augment the electrical signals, which showed a great performance in boosting the classification accuracy.

In summary, the main contributions of our present work are:

1. Our work established SWP as an effective parameter that can be utilized to differentiate plant local and systemic wound responses when they are deprived of light at different levels.
2. New features were established for efficiently classifying plant electrical signals. In our case, the electrical signals were collected from wounded plants that were pretreated under darkness to different extents.
3. An improved Adversarial Autoencoder (AAE) structure was proposed to augment the electrical signals.

The following sections of the manuscript were organized as below: (1) Materials and Methods. In this section, the pipeline for the data acquisition, preprocessing, feature extraction, data augmentation and classification models were described in detail. (2) Results. This section presented the classification results of SWPs as well as performance evaluation of our proposed data augmentation structure. (3) Discussion. The results and the limitations of the present work were discussed in this Sect. (4) Conclusion. This section summarized the overall findings and potential future applications.

## Materials and methods

### Plant growth conditions

**Fig. 1:**
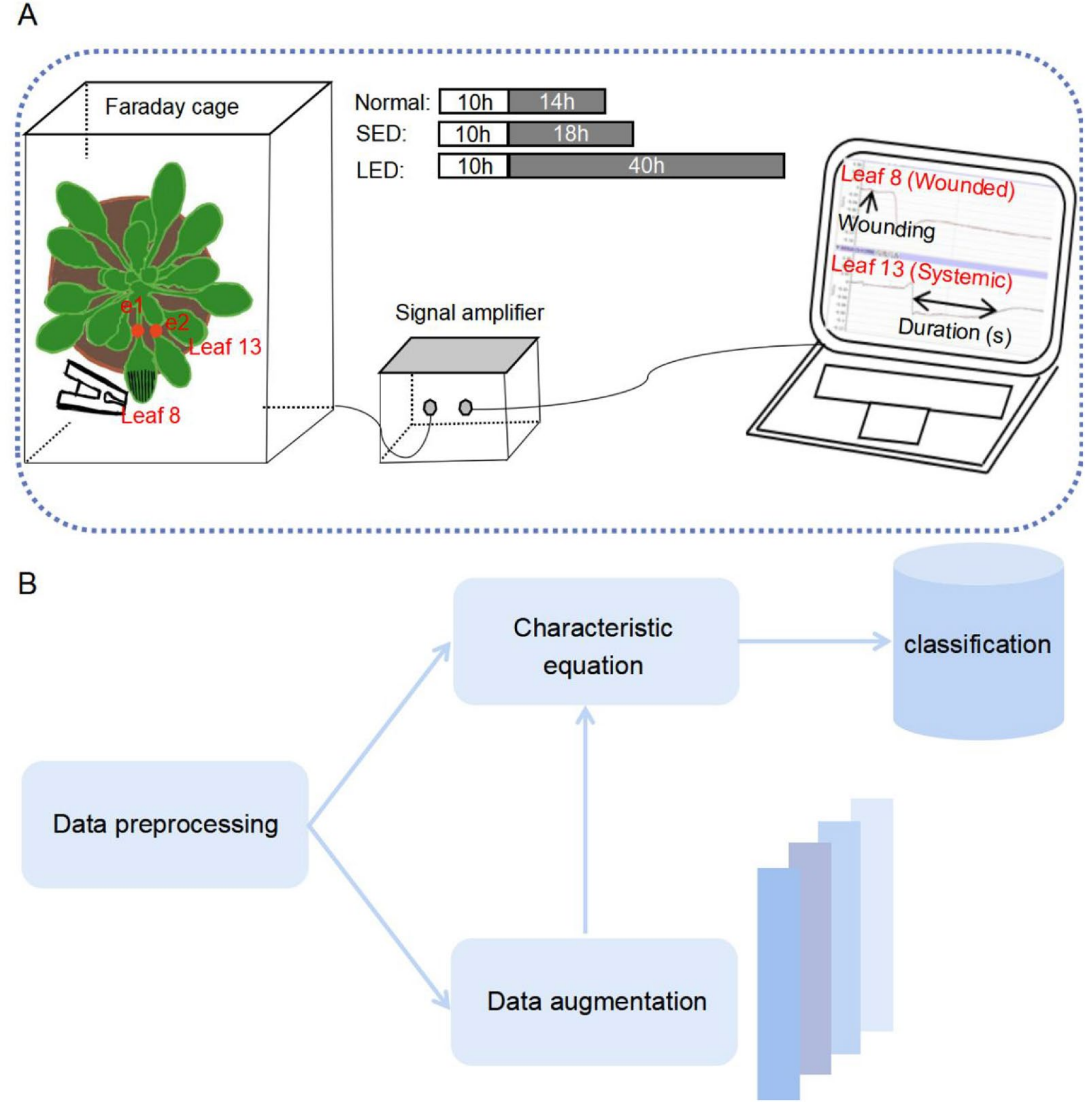
Pipeline for the experimental setup and the data analysis. **A** Overview of the data acquisition system. To avoid external interference, 5-week-old *Arabidopsis* plants were treated under different light conditions before measurement in a Faraday cage. Light conditions: Normal: 10 h light/14 h night; SED: short extended darkness, 10 h light/18 h night; LED: long extended darkness, 10 h light/40 h night. Leaf 8 was wounded with forceps and the electrical signals were measured by two electrodes (e1, e2) that were placed on the petioles of leaf 8 and leaf 13, respectively. The signals were amplified through a signal amplifier and then converted for visualization on the laptop as shown. **B** Overall workflow for classifying the electrical signals. Following preprocessing, characteristics were extracted from the leaf 8 and leaf 13 electrical signals, respectively. Machine learning-based classification was performed using either the extracted characteristics from original samples or extended samples upon data augmentation. The colored bars next to “Data augmentation” represent the structural network for data augmentation

5-week-old *Arabidopsis thaliana* Colombia-0 (Col-0) plants were used as a model in this study. Individual plants were grown in the growth room at 21 °C under 150 μE m^−2^ s^−1^ light (10 h light, 14 h dark, 70% humidity) until 5-week-old with expanded rosettes. Then the plants were subjected to darkness treatment before measuring wound-induced electrical signals. Normal light condition: 10 h light/14 h night. Two extended darkness conditions were introduced: (1) short extended darkness (SED, 10 h light/18 h night); (2) long extended darkness (LED: 10 h light/40 h night). The above three conditions are summarized in Fig. 1A.

### Experimental setup and data acquisition

As shown in Fig. 1A, measurements of wound-induced SWPs were carried out in a Faraday cage to avoid interference. Two Ag/AgCl recording electrodes (e1, e2) were placed on the petioles of leaf 8 (wounded) and leaf 13 (systemic), respectively. One drop of KCl/agar solution was added in between the electrode and the leaf petiole to maintain the connection. Another Ag/AgCl electrode was placed in soil as a reference. Upon crushing leaf 8 (wounded), the signals from both e1 and e2 were collected at a frequency of 100 Hz, amplified through a high-impedance signal amplifier (FD223a, WPI), and finally visualized using LabScribe3 software (iWorx System, Inc., Dover, NH). In this study, we collected SWPs from 20 plants under normal light conditions, 20 plants treated with SED and 15 plants treated with LED. For each plant, leaf 8 and leaf 13 SWPs were separately measured. In Table 1, the total number of the measured plants under different light conditions is listed.

**Table 1:** Number of samples measured before treating with different light conditions

Leaves	Treatments	Number of samples
Leaf 8	Normal	20
	SED	20
	LED	15
Leaf 13	Normal	20
	SED	20
	LED	15

Electrical signals from both leaf 8 (wounded) and leaf 13 (systemic) were collected

### Overall framework of data analysis

Figure 1B shows the overall workflow for analyzing the SWPs in this study. It contains the following procedures: (1) data preprocessing; (2) obtaining of characteristic equations; (3) data augmentation; (4) classification using different models.

### Data preprocessing

#### Signal extraction

**Fig. 2:**
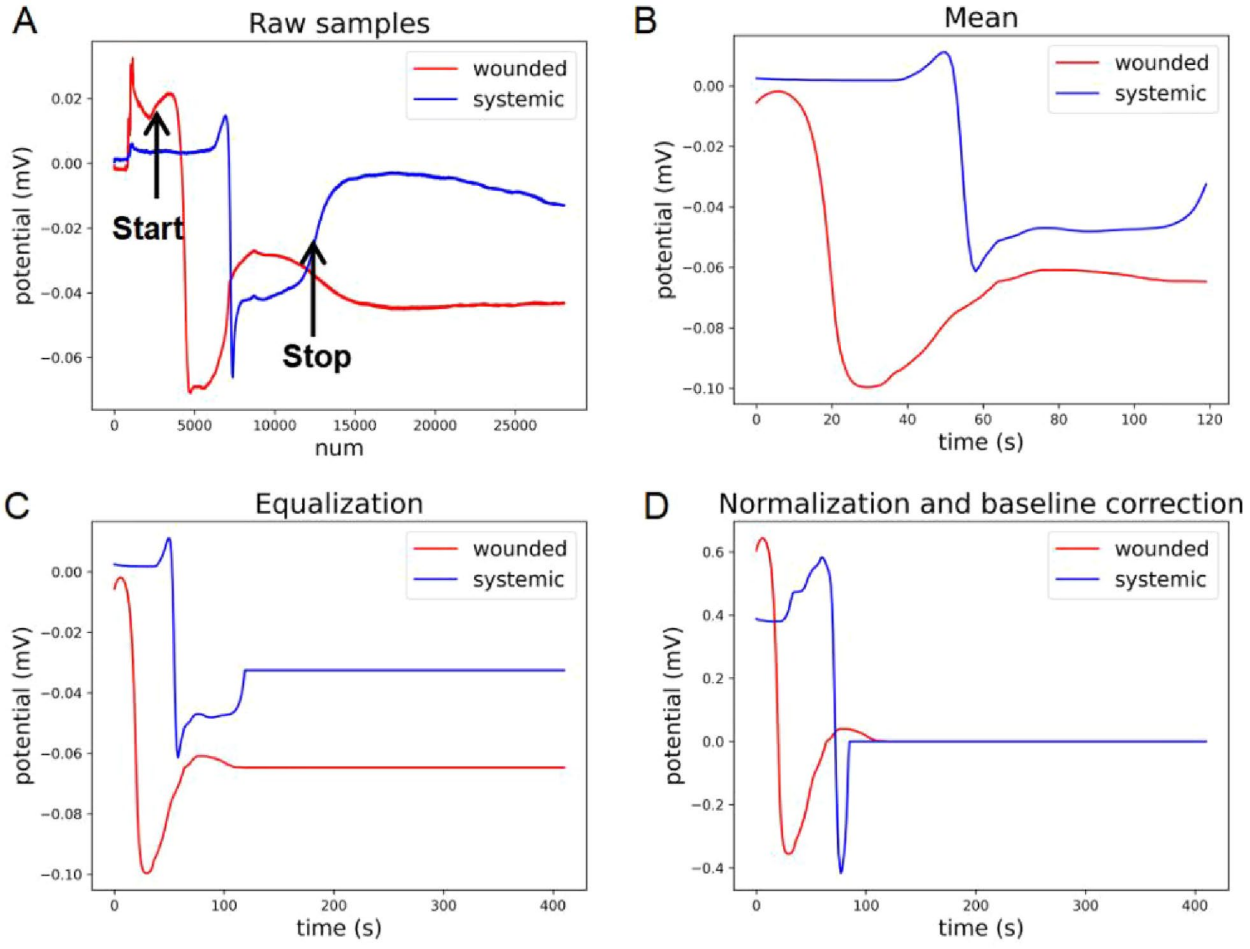
Data preprocessing. **A** The electrical signal between the “start” and “stop” window was extracted from each raw recording as the useful signal. The horizontal axis shows the number (num) of the data points within the sample. **B** The values of every 100 data points in the window of **A** were averaged and the resulting mean trace is shown. The horizontal axis was converted to show the duration (s) within the window. **C** The mean trace in **B** was equalized with a length of 411 s by filling the sample with the value of the last data point. **D** Normalization and alignment of the equalized trace in **C**

It was well established in the previous study [7] that when a local leaf (usually leaf 8) of *Arabidopsis* plants is wounded, SWPs could be generated in this leaf and then successfully transmitted to the distal part of plants. Leaf 13, which is distal to leaf 8, is normally measured to assess the systemic defense responses reflected by the SWP signals. Therefore, in our study, we also focused on the SWPs from this pair of leaves to estimate the local and systemic wound responses of the plants. As SWPs in the local leaf typically did not recover to the baseline during recording, and the time point for wounding the plants varies from sample to sample, to facilitate comparable features of SWPs under different conditions, we extracted electrical signals from each sample following the same criteria. Specifically, electrical signals used for further analysis were extracted in between the “start” and “stop” window of the raw sample (as shown in Fig. 2A). Start: the time point when wounding was applied. Stop: the time point when the repolarization phase recovers to half of the maximum depolarization. For each sample, all the data points in this window were extracted and represented the useful signals. In the example trace in Fig. 2A, there are 12,800 data points after the signal extraction (between “start” and “stop”).

#### Local mean compression

The process of local mean compression was performed on the extracted leaf 8 and leaf 13 signals for a specific purpose. The main objective was to reduce the amount of data while preserving the essential features of the original signals. Specifically, the signals were first divided into smaller segments. Then the mean values were calculated for each segment and were used to represent the entire segment. The compressed signal will have fewer data points compared to the original signal while the mean values of each segment still retain the essential features of the original signal. In this study, as shown in Fig. 2B, we set a fixed-size sliding window with a width of 100 data points. Following this, the original 12,800 data points were converted to a duration of 128 s (12,800/100 = 128). The same treatment was applied to all the samples.

#### Padding

Many machine learning models, especially those based on neural networks, require inputs of fixed dimensions. In this study, the duration of electrical signals can differ from one sample to another. Padding [25] was performed to fix the inputs into the same dimension. To do so, the samples were all filled with the values of their last data points to ensure an equal duration of 411 s, which corresponded to the sample with longest duration (Fig. 2C).

#### Normalization and baseline correction

We normalized each sample of all datasets to [− 1,1] by Eq. (1), which allows variables with uncertainties to become more comparable. Normalization will make the model more data-sensitive which improves the accuracy of classification [26].

1 $$Y_{i} = \frac{{X_{{i,j}} - X_{{i,min}} }}{{X_{{i,max}} - X_{{i,max}} }}{\text{~~}}\left( {i = 1,2, \cdots ,n;{\text{~~}}j = 1,2, \cdots ,411} \right)$$

where ${Y}_{i}$ is the $i$-*th* normalized sample, ${X}_{i,j}$ is the $j$-*th* datapoint of the $i$-*th* sample, ${X}_{i,min}$ is the minimum datapoint of the $i$-*th* sample, ${X}_{i,max}$ is the maximum datapoint of the $i$-*th* sample, $i$ is the number of samples and $j$ is the number of datapoints.

Next, to reduce the impact of the filled values on the following calculations, they were aligned to the amplitude at “0”. The details of this section are shown in Fig. 2D.

### Feature extraction

#### Time domain characteristics

To classify wound-induced Leaf 8 and Leaf 13 SWPs from plants that were treated with three light conditions (Normal, SED and LED), we started with extracting 12 time-domain features [27] from the SWP signals (Additional file 1: Table S1). Maximum and minimum respectively represent the maximum and minimum potential values in a sample. The mean is computed by dividing the overall potential values by the number of data points in a sample. The variance reflects the degree of fluctuation of the signal and is calculated by the distance between the potential value and the mean value at each time point. The standard deviation is the root of the variance. The skewness measures the skewed direction and degree of signal distribution. The kurtosis measures the sharpness of the signal distribution. The mean square is defined as the average value of each potential value squared. The root mean square is the root of the mean square value. The area represents the overall trend of a useful signal over the entire period. The declining slope represents the slope of the signal from the first maximum value to the minimum value. The rising slope represents the slope of the signal from the minimum value to the next maximum value. The peak represents the difference between the maximum and the minimum peak values. The equations for calculating the above features are computed below:

**Table:** 

${{\text{Maximum}}=max(x}_{i})$	${Minumum=min(x}_{i})$	(2)
${\text{Mean}}:=\frac{1}{ n}{\sum }_{i=1}^{n}{x}_{i}$	${Variance: \sigma }^{2}=\frac{1}{n}{\sum }_{i=1}^{n}{({x}_{i}-\overline{x })}^{2}$	
$\mathrm{Standard\, deviation}:\mathrm{ S}=\sqrt{{\upsigma }^{2}}$	$Kurtosis: K=\frac{\frac{1}{n}\sum_{i=1}^{n}{({x}_{i}-\overline{x })}^{4}}{{[\frac{1}{n}\sum_{i=1}^{n}{({x}_{i}-\overline{x })}^{2}]}^{2}}$	
${Mean\, square\, root:x}_{rms}=\sqrt{\frac{1}{n}{\sum }_{i=1}^{n}{|{x}_{i}|}^{2}}$	${\text{A}}rea={\sum }_{i=1}^{n}|\frac{(\left(f\left({x}_{i}\right)+f\left({x}_{i-1}\right)\right)*({x}_{i+1}-{x}_{i}))}{2}|$	
$\mathrm{Decline\, slope}=\frac{maximum-minimum}{-\Delta t}$	$\mathrm{Rising slope}\hspace{0.17em}=\hspace{0.17em}\frac{maximum-minimum}{\Delta t}$	
$\mathrm{Amplitude }={max(x}_{i})-{min(x}_{i}$)	${\text{Skewness}}\hspace{0.17em}=\hspace{0.17em}\frac{\frac{1}{n}{\sum }_{i=1}^{n}{({x}_{i}-\overline{x })}^{3}}{{[\frac{1}{n}\sum_{i=1}^{n}{({x}_{i}-\overline{x })}^{2}]}^{3/2}}$	

Where ${x}_{i}$ represents the $i$-*th* datapoint in a sample, $i=\mathrm{1,2},\cdots 411$.

#### Derivative and integral transformation

Plant electrical signals are kinds of time series, and are non-stationary and nonlinear. As a mathematical method, derivative transformation calculates the derivative of a signal and can be used to isolate specific features in a signal, such as edges, spikes, and changes in slopes [28]. The integral of a signal represents the accumulated change of the signal over time [29]. As another mathematical method, integral transformation calculates the integral of a signal. It is advantageous in signal smoothing, noise eliminating, and information extracting from the overall shape and trends of signals. Both derivative and integral transformation facilitate analysis and characterization of the accumulated change in a signal over time. The formulas for computing the first-order derivative and the integral are given in Eq. (3). The first-order derivative (deriv_1st) and the integral of a representative leaf 8 (wounded) SWP under normal condition are shown in Additional file 1: Fig. S1.

3 $${{\text{f}}}^{\mathrm{^{\prime}}}\left({{\text{x}}}_{{\text{i}}}\right)=\frac{1}{2}(\frac{{{\text{y}}}_{{\text{i}}+1}-{{\text{y}}}_{{\text{i}}}}{{{\text{x}}}_{{\text{i}}+1}-{{\text{x}}}_{{\text{i}}}}+\frac{{{\text{y}}}_{{\text{i}}}-{{\text{y}}}_{{\text{i}}-1}}{{{\text{x}}}_{{\text{i}}}-{{\text{x}}}_{{\text{i}}-1}})\mathrm{ s}=\frac{\left({\text{f}}\left({{\text{x}}}_{{\text{i}}+1}\right)+{\text{f}}({{\text{x}}}_{{\text{i}}})\right)*({{\text{x}}}_{{\text{i}}+1}-{{\text{x}}}_{{\text{i}}})}{2} ({\text{i}}=\mathrm{1,2},\cdots ,411.)$$

where ${x}_{i}$ represents the $i$-*th* datapoint in a sample, $i=\mathrm{1,2},\cdots 411$.

### Data augmentation

The core concept of few-shot learning lies in addressing the issue of limited samples, which often impacts classification accuracy due to experimental constraints [30]. To enhance the model’s generalization ability and subsequently improve classification accuracy, one approach is to expand the number of electrical signal samples by generating augmented data within the training sets. In our study, sample augmentation for plant electrical signals based on Adversarial Autoencoder (AAE) [31] was proposed. AAE was shown as a powerful tool in several aspects like semi-supervised classification, unsupervised clustering, image generation and data visualization [32]. However, its application in augmenting plant electrical signal samples has not been explored until now. This innovative use of AAE presents a significant opportunity to bolster few-shot learning capabilities in the context of plant electrical signal analysis.

#### Architecture for the data augmentation

The autoencoder (AE) [33] is composed of an encoder and a decoder. The encoder extracts key informative features from high-dimensional original data and maps them to a low-dimensional latent representation. Then the decoder maps the latent representation back to the original high-dimensional input. AAE adds a discriminator to the AE. After the original data is mapped to the latent space, the data conforming to a specific distribution is input. The discriminator is used to determine whether the mapped data space follows a specific data distribution pattern.

**Fig. 3:**
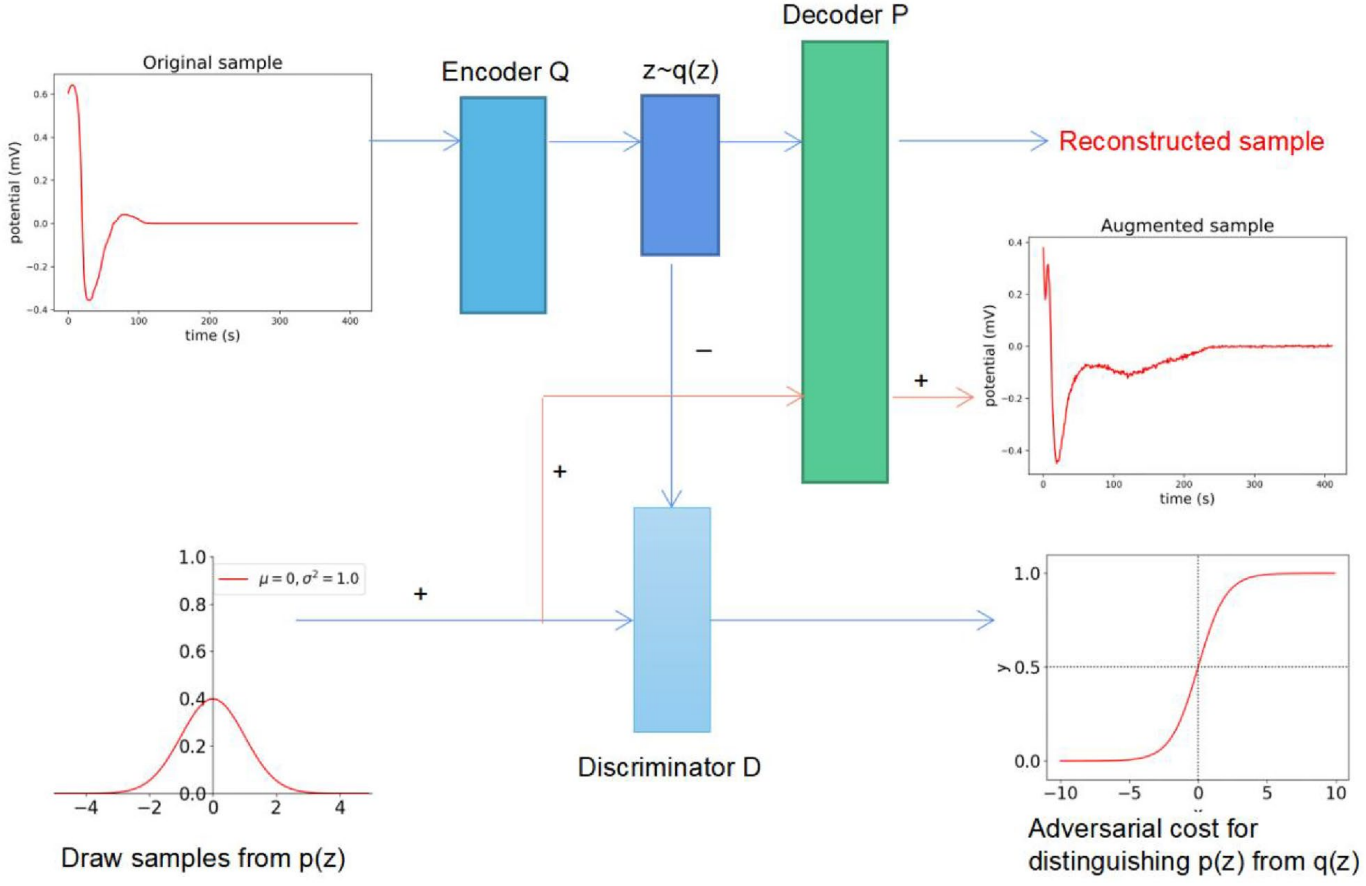
Pipeline of the data augmentation.’ + ’ represents the virtual vector,’-’ represents the real vector. The pipeline consists of Encoder Q, Decoder P and Discriminator D. The curve in the lower left corner is a Gaussian distribution plot. The curve in the lower right corner shows a sigmoid function

As shown in Fig. 3, the entire network structure is composed of the autoencoder (upper) and the discriminator (lower). The training process is divided into two stages: in the sample reconstruction stage, the original samples were input as one-dimensional data. The autoencoder was renewed by Stochastic Gradient Descent (SGD) [34] to minimize the reconstruction loss function. Here, we proposed to use the L1 loss function given by Eq. (5). Regularization [35] is accomplished using the aggregated posterior ${\text{q}}\left({\text{z}}\right)$ to match any prior ${\text{p}}({\text{z}})$ in the regularization phase. The encoding function $q({\text{z}}|{\text{x}})$ on the autoencoder defines the hidden encoding ${\text{q}}({\text{z}})$, and the vector aggregated posterior distribution is computed as:$q\left(z\right)={\int }_{{\text{x}}}^{ }{\text{q}}({\text{z}}|{\text{x}}){{\text{p}}}_{{\text{d}}}({\text{x}}){\text{dx}}. P(z)$ is the data that conforms to a certain distribution. Here we set it as the data that conforms to Gaussian distribution, which alternately allows updating the discriminant network and generator. We proposed to use Generative Adversarial Network [36] to complete the network training. The idea of using it is shown below:

4 $$\begin{array}{c}{\text{min}}\\ {\text{G}}\end{array}\begin{array}{c}{\text{max}}\\ {\text{D}}\end{array}{{\text{E}}}_{{\text{Z}}\sim {{\text{p}}}_{{\text{data}}}}[{\text{logD}}({\text{p}}({\text{z}}))+{\text{log}}(1-{\text{D}}({\text{G}}({\text{q}}({\text{z}}))))]$$

where $p(z)$ is the real Gaussian data distribution. $G(q(z))$ is the virtual data distribution generated according to the input samples. $D(p(z))$ represents the probability of $D$ judging the real data distribution. $D(G(q(z)))$ represents the probability of $D$ judging the distribution of virtual data.

5 $${\text{L}}1\_{\text{loss}}:\mathrm{ L}1\left(\widehat{{\text{y}}}-{\text{y}}\right)={\sum }_{{\text{i}}=0}^{{\text{n}}}|{\widehat{{\text{y}}}}^{({\text{i}})}-{{\text{y}}}^{({\text{i}})}|$$

where $\widehat{y}$ represents the predicted value, $y$ represents the real value, and $i$ represents the sample dimension.

The encoder, decoder, and the discriminator are all composed of fully-connected layers. The encoder contains 5 linear layers. Considering that each sample has 411 data points, the number of input neurons was set to 411, and the number of output neurons is 50. Corresponding to the encoder, the decoder also consists of 5 linear layers, with 50 input neurons and 411 output neurons. The discriminator consists of five linear layers with 50 input neurons and 1 neuron as a discriminative output. Detailed network architecture is shown in Additional file 1: Fig. S2. Dropout [37] was set to 0.2 for the linear layer to prevent overfitting. Relu activation function [38] was used to better mine sample features. fivefold cross-validation was used to split the dataset. The remaining parameters were set as follows: seed = 10, epoch = 200, reconstruction learning rate reg_lr = 0.0001, and generation learning rate gen_lr = 0.0005.

#### Evaluation criterion for the data augmentation

To evaluate the generative AAE, the mean values of each dimension from all samples were used as the template, and the similarity between the original sample and the augmented sample was compared using the following four indicators:

Euclidean distance (ED) measures the distance between two points in a multidimensional space.

6 $$\mathrm{ ED}=\frac{{\sum }_{h=1}^{{\text{m}}}\sqrt{{\sum }_{{\text{i}}=1}^{{\text{n}}}{({{\text{x}}}_{{\text{i}}}-{{\text{y}}}_{{\text{i}}})}^{2}}}{{\text{m}}}$$

Pearson correlation coefficients (PCC) measures the levels of correlation between $x$, $y$ variables.

7 $${\text{PCC}}=\frac{1}{{\text{m}}}\left({\sum }_{h=1}^{{\text{m}}}\frac{{\sum }_{{\text{i}}=1}^{{\text{n}}}\left({{\text{x}}}_{{\text{i}}}-\overline{{\text{x}} }\right)\left({{\text{y}}}_{{\text{i}}}-\overline{{\text{y}} }\right)}{\sqrt{{\sum }_{{\text{i}}=1}^{{\text{n}}}{\left({{\text{x}}}_{{\text{i}}}-\overline{{\text{x}} }\right)}^{2}}\sqrt{{\sum }_{{\text{i}}=1}^{{\text{n}}}{\left({{\text{y}}}_{{\text{i}}}-\overline{{\text{y}} }\right)}^{2}}}\right) \overline{\mathrm{x } }={\sum }_{{\text{i}}=1}^{{\text{n}}}{{\text{x}}}_{{\text{i}}} \overline{{\text{y}} }={\sum }_{{\text{i}}=1}^{{\text{n}}}{{\text{y}}}_{{\text{i}}}$$

Cosine similarity (CS) indicates the similarity between two vectors by measuring the cosine of the angle.

8 $$\mathrm{cos\theta }=\frac{1}{{\text{m}}}({\sum }_{h=1}^{{\text{m}}}\frac{{\sum }_{{\text{i}}=1}^{{\text{n}}}({{\text{x}}}_{{\text{i}}}*{{\text{y}}}_{{\text{i}}})}{\sqrt{{\sum }_{{\text{i}}=1}^{{\text{n}}}{{\text{x}}}_{{\text{i}}}^{2}}\sqrt{{\sum }_{{\text{i}}=1}^{{\text{n}}}{{\text{y}}}_{{\text{i}}}^{2}}})$$

where $m$ is the number of samples, $n$ is the number of dimensions,${x}_{i}$ represents the dimension of the original sample or the augmented sample, ${{\text{y}}}_{{\text{i}}}$ represents the dimension of the template.

If the similarity between the original sample and the template is close to that between the augmented sample and the template, the augmented sample is considered reliable.

### Classification

#### Classifier

Support Vector Machine (SVM) [39]. SVM is a supervised learning algorithm used for classification and regression tasks. It is a powerful and versatile classifier that finds the optimal hyperplane to separate data into different classes. In the SVM model, C is the regularization parameter, which is crucial in avoiding overfitting or underfitting issues. In our case, the value of C was set to 1 to balance the model’s complexity and fitting capability. To deal with non-linearly separable data, we used the ‘RBF’ (Radial Basis Function) kernel as the kernel function. In the RBF kernel function, gamma is an important parameter that determines the impact of each sample on the decision boundary. We chose to automatically adjust the gamma value to determine the optimal decision boundary based on the characteristics of the data. By optimizing the values of C and the parameters for the kernel function, SVM is expected to achieve a good performance in classification problems, in our case, in classifying wound-induced SWPs from plants treated with different light conditions.

K-Nearest Neighbor (KNN) [40]. The KNN algorithm follows a specific classification process. First, it calculates the distances between each testing sample and all other samples in the dataset. Then the distances are sorted, and the k samples with the shortest distances to the testing sample are selected. Next, the categories of these k-nearest samples are determined. Finally, the testing sample is classified based on the category that is most frequently represented among the k-chosen neighbors. By considering multiple nearest neighbors, the KNN algorithm leverages the collective information from neighboring samples to make a more informative and reliable classification decision. This approach enhances the accuracy and robustness of the classification results. In our classification process, we tested different values of k to assess the impact on classification accuracy. After evaluation, we determined to set the value of k to 5, which resulted in the best classification accuracy.

Random Forest [41]. Random Forest is a commonly used supervised learning algorithm composing of multiple decision trees. The "random" refers to two key components. First, when constructing each decision tree, a random subset of samples is selected from the original dataset using sampling with replacement. Second, a random subset of features is selected as input variables, which helps to increase the diversity among decision trees and reduce the risk of overfitting. By setting random_state = 0, the results of running the Random Forest algorithm are reproducible. In other words, the same random seed will lead to the same random sampling and feature selection results, therefore ensuring consistency and comparability of the output results. The classification result of Random Forest is determined by a voting mechanism. After the samples are classified by each decision tree, the final classification is determined based on the voting results of all decision trees. This ensemble learning approach helps to improve the accuracy and robustness of the Random Forest algorithm.

Muti-Layer Perception (MLP) [42]. Multi-layer perceptron (MLP) is a classical neural network model. It usually consists of an input layer, a hidden layer and an output layer. The different layers are fully connected. In our study, we incorporated this deep learning model, in addtion to the other machine learning models described above, to evaluate their performance in SWP classification. The number of hidden layer neurons is set to 10. Relu is used as the activation function between layers. The MLP model is ended up with Softmax layer, which outputs the confidence of each category. Learning_rate = 0.01.

#### Evaluation criterion for the classifiers

To visualize the classification results, we introduced confusion matrix with the horizontal axis representing the true label and the vertical axis representing the predicted label.

The confusion matrix contains four pieces of information: True Positive (TP), which means the actual positive samples are predicted to be positive; True Negative (TN), which means the actual negative samples are predicted to be negative; False Positive (FP), which means the actual samples are negative but predicted to be positive; False Negative (FN), which means the actual samples are positive but predicted to be negative.

The following four indicators were used to evaluate the classification effect:

**Table:** 

${\text{Precision}} = \frac{{{\text{TP}}}}{{{\text{TP}} + {\text{FP}}}}$	${\text{Recall}} \,=\, \frac{{{\text{TP}}}}{{{\text{TP}} + {\text{FN}}}}$	(9)
F1 score = $2*\frac{{\text{Precision}}\,*\,{\text{Recall}}}{{\text{Precision}}\,+{\text{Recall}}}$	${\text{ACC}}\, = \,\frac{{{\text{TP}} + {\text{TN}}}}{{{\text{TP}} + {\text{TN}} + {\text{FP}} + {\text{FN}}}}$	

Precision represents the proportion of samples that are actually positive among the samples that are predicted to be positive. The recall is determined as the ratio between the number of positive samples that are correctly classified as positive to the total number of positive samples. The recall measures the model's ability to detect positive samples. The higher the recall, the more positive samples detected. F1 score is calculated as a weighted average of precision and recall. Greater F1 score reflects robuster model. Given that our task is a multi-classification, we used macro-averaging method that first calculated the precision, recall, and F1 score for each positive example and then averaged all the results. Accuracy (ACC) represents the proportion of the correctly classified samples out of the total number of samples.

## Results

Leaf 8 and leaf 13 SWPs separately evaluate the local and systemic wound responses for a plant. In this study, plants were pre-treated under either normal or extended darkness conditions (SED and LED) before leaf 8 and leaf 13 SWPs were measured upon wounding leaf 8. Then leaf 8 and leaf 13 signals were separately classified using various models. In this section, we presented the triple classification results for leaf 8 (wounded) and leaf 13 (systemic) SWPs, respectively. To improve the classification accuracy, we introduced a new data augmentation network to extend the sample size for the electrical signals. The performance of this method was evaluated in comparison to another two widely used methods.

### Triple classification of wound-elicited SWPs under extended darkness using original data

After performing the data preprocessing procedures shown in **Materials and Methods** “Data preprocessing” on leaf 8 (wounded) and leaf 13 (systemic) SWP samples respectively, we extracted a group of 12 time-domain characteristics, the first-order derivative and the integral features from the traces. The features were then subjected to classification using SVM, KNN and Random Forest, and MLP classifiers, respectively. Triple classification results for leaf 8 (wounded) and leaf 13 (systemic) SWPs are shown in Additional file 1: Tables S2, S3, respectively. Table 2 shows the best classification results. In the case of leaf 8 (wounded) SWPs, an accuracy of 0.89 was reached using the integral as the feature and SVM as the classifier. For classifying leaf 13 (systemic) SWPs, the SVM classifier showed the best performance and generated a classification accuracy of 0.8 when using the integral as the feature. Details of the classification are shown in the confusion matrices in Additional file 1: Fig. S3. Six samples for leaf 8 SWPs were misclassified (Additional file 1: Fig. S3A) while eleven samples were misclassified for leaf 13 SWPs (Additional file 1: Fig. S3B).

**Table 2:** The best classification results for leaf 8 (wounded) and leaf 13 (systemic) SWPs

Leaves	Features	Classifiers	Accuracy	Recall	Precision	F1 score
Leaf 8	Integral	SVM	0.89	0.89	0.90	0.89
Leaf 13	Integral	SVM	0.80	0.80	0.80	0.80

### Evaluation of the augmented samples

To improve the classification accuracy for the misclassified leaf 8 and leaf 13 samples as shown in Additional file 1: Fig. S3, we proposed to use a data augmentation strategy to generate augmented leaf 8 and leaf 13 samples for training the models. After preprocessing the samples as shown in **Materials and Methods** “Data preprocessing”, the samples were augmented using AAE as described in the methods. Four evaluation indicators described in **Materials and Methods** “Evaluation criterion for the data augmentation” were used to evaluate the similarities between the original and the augmented samples. The mean values of each dimension of the original sample under Normal, SED and LED conditions were calculated and used as templates. For evaluation, we used the ED, CS and PCC parameters to compare the correlation among the templates, the original samples and the augmented samples. As shown in Table 3, for both leaf 8 and leaf 13 SWPs, the Euclidean distance between the augmented sample and the template, and that between the original sample and the template, is very close. In addition, the PCC and CS values are close to 0.8, indicating that the templates are strongly correlated to all the samples. Together, our results demonstrated that the augmented samples are very similar to the original samples, supporting the effectiveness of our proposed data augmentation model.

**Table 3:** Quality evaluation of the augmented samples for leaf 8 (wounded) and leaf 13 (systemic) SWPs

Leaves	Indicators	Augmented Normal_template	Original Normal_template	Augmented SED_template	Original SED_template	Augmented LED_template	Original LED_template
Leaf 8	ED	11.51	14.21	14.99	14.39	20.06	13.09
	CS	0.83	0.75	0.83	0.75	0.94	0.88
	PCC	0.82	0.74	0.81	0.72	0.93	0.87
Leaf 13	ED	17.76	17.81	23.16	24.82	23.82	16.43
	CS	0.84	0.78	0.94	0.82	0.83	0.79
	PCC	0.84	0.77	0.89	0.78	0.79	0.79

### Triple classification of wound-elicited SWPs under extended darkness upon data augmentation

Based on the above model, 50 augmented samples were respectively generated for leaf 8 and leaf 13 SWPs under normal, SED and LED conditions. Similarly, we extracted the features and reclassified the leaf 8 and leaf 13 SWPs using the above four classifiers with the same settings as for the original data. For all of the datasets, the leave-one-out method was used to divide the training and testing sets. Upon data augmentation, the classification results were generally improved in all the combinations of different features and classifiers as reflected by the four parameters (Additional file 1: Table S4 and Table S5). We noted that upon adding the augmented samples, Random Forest showed the best performance in classifying either leaf 8 or leaf 13 SWPs using their deriv_1st as features (Table 4).

**Table 4:** The best classificaiton results for leaf 8 (wounded) and leaf 13 (systemic) SWPs upon data augmentation

Leaves	Features	Classifiers	Accuracy	Recall	Precision	F1 score
Leaf 8	Deriv_1st	Random forest	0.76/0.99	0.76/0.99	0.77/0.99	0.76/0.99
Leaf 13	Deriv_1st	Random forest	0.71/0.99	0.69/0.99	0.75/0.99	0.70/0.99

The results before augmentation are shown before slash

Specifically, the classification accuracy for leaf 8 (wounded) SWPs was increased to 0.99 when using the deriv_1st as the feature and Random Forest as the classifier (Table 4). Moreover, the scores for other evaluation indicators (Recall, Precision, F1-score) were also greatly boosted (Table 4). Detailed classification results are shown in the confusion matrix in Additional file 1: Fig. S4. Originally, 13 samples were misclassified (Additional file 1: Fig. S4A), whereas, upon data augmentation, only two samples were incorrectly classified under SED and LED conditions (Additional file 1: Fig. S4B).

For leaf 13 (systemic) SWPs, as shown in Table 4, the best classification accuracy was also greatly elevated to 0.99. Similar to leaf 8 SWPs classification, Random Forest again showed the best performance when using the deriv_1st as the feature. Detailed information for the classification was shown in Additional file 1: Fig. S4. Before data augmentation, seven samples under normal condition, two samples under SED condition and seven samples under LED condition were misclassified (Additional file 1: Fig. S4C) whereas after data augmentation, only one sample under normal condition and one sample under SED condition were misclassified (Additional file 1: Fig. S4D).

### Performance comparison of the different augmentation methods

Finally, to evaluate if our proposed augmentation method was superior to other reported models, we compared the performance of AAE to the Generative Adversarial Network (GAN) [36] and the Variational Autoencoder (VAE) models [43] in augmenting SWPs. The GAN method was proposed to enrich the electrical signals from wheat seedlings under salt tolerance. By introducing a certain amount of noise into the model, a regularization effect was produced and the performance of the model was improved. The classification accuracy was increased from 80.77 to 92.31% [18]. Variational Auto-Encoder (VAE) is a generative algorithm which is highly versatile and its function is not affected by the format of data [44]. However, it has not been applied in generating plant electrical signals before.

To facilitate comparison, the same signal preprocessing, feature extraction and classifying procedures were used before deploying the GAN and VAE methods. The dataset was divided into training set and testing set by fivefold cross-validation. All experiments were done by CPU through python3.7 under win10 operating system. In the case of using GAN to generate SWPs, we reused the same settings reported in [18], to compare its performance in augmenting and classifying our electrical signals with our proposed AAE. The number of input and output neurons was set to 411 based on the model proposed in [18]. In the case of using VAE to generate SWPs, as it was not reported in augmenting electrical signals before, the parameters were set the same as those for AAE. Specifically, the encoder consists of five linear layers with each layer containing 411, 200, 100, 100, and 50 neurons, respectively. The decoder consists of five linear layers with each layer containing 50, 100, 100, 200, and 411 neurons, respectively.

As shown in Table 5, in comparison to GAN and VAE, our proposed AAE method showed the best performance in combination with the tested classifiers to classify leaf 8 and leaf 13 SWPs. Altogether, the results strongly suggest that our method is an effective tool for augmenting SWPs in the testing sets, which further allowed us to improve the performance of the classifiers.

**Table 5:** Comparison of the classification accuracies (ACC (%)) for leaf 8 (wounded) and leaf 13 (systemic) SWPs using three different augmentation methods

Leaves	Time-domain (ACC)			Deriv_1st (ACC)			Integral (ACC)		
	AAE	VAE	GAN	AAE	VAE	GAN	AAE	VAE	GAN
Leaf 8	82.44	79.51	84.39	95.61	89.75	67.32	95.61	89.26	83.90
	80.98	82.43	81.95	90.24	87.31	69.93	84.88	85.85	86.83
	80.49	81.95	81.95	**99.02**	90.24	80.49	91.22	88.78	84.88
Leaf 13	87.80	64.88	76.10	93.66	89.27	76.59	97.07	91.71	84.39
	88.78	77.56	76.59	86.83	88.78	71.22	91.21	91.21	84.39
	88.78	84.39	80.49	**99.02**	90.73	70.73	96.10	93.65	83.90

The best results are underlined and highlighted in bold

## Discussion

Plants can generate electrical signals when subjected to various environmental stimuli or grown at different developmental stages [45, 46]. Particularly, in recent years, wound-induced electrical signals, widely known as slow wave potentials (SWPs), are receiving great attention as they were found to tightly connect with plant defense activities in response to herbivory or mechanical damage [45]. Therefore, decoding SWPs under different conditions will potentially enable differentiating whether plants could react differently to mechanical stress when grown under a varying environment. In this work, we measured local (leaf 8) and systemic (leaf 13) wound-induced SWPs from *Arabidopsis* plants that were kept in darkness to different extents (normal, short-extended darkness and long-extended darkness) and classified the signals in combination with machine learning models. Extended darkness has a great impact on plant growth [23]. Moreover, in a recent study by Fotouhi et al. [47], it was shown that extended darkness led to a failure in generating SWPs in an *aca10 aca12* mutant, which subsequently enhanced the susceptibility to herbivory. By extracting different features from SWPs and applying different machine learning models, we managed to classify leaf 8 and leaf 13 SWPs separately from plants treated under darkness to different extents (SED and LED) compared to normal light condition, with accuracies above 0.8. To further improve the performance, we introduced an AAE method to augment our obtained SWP samples. The classification accuracies were greatly increased up to more than 0.99 for both leaf 8 and leaf 13 SWPs. Our work established SWPs as an effective proxy that can be applied to differentiate plant local and systemic wound responses when they are deprived of light to different extents. This is actually in line with the genetic evidence that extended darkness reshapes plant wound responses, for example, in the *aca* mutants [47]. Therefore, our proposed methods for classifying SWPs under different light conditions could be further applied in the early detection of various wound responses in different genetic mutants when they are subjected to different environmental stimuli.

From the perspective of methodology, we introduced to use the integral and the deriv_1st as new features for plant electrical signals. Compared to the canonical duration and amplitude features used to quantify electrical signals [45], our established parameters also effectively allowed distinguishing plant wound response-SWPs affected by different levels of darkness. However, accurate and reliable classification of plant electrical signals under different conditions is still counteracted by the limited number of samples. Although data augmentation has been implemented as a useful tool to improve the performance of models [48, 49], its application in augmenting plant electrical signals was poorly investigated. In our work, we proposed to use AAE method to generate virtual SWP samples for training the machine learning models. This method has not been reported in augmenting plant electrical signals before. We compared the classification results using data obtained by three different data extension methods, out of which, our proposed AAE model obtained the best result for classifying SWPs. This is probably because the AAE idea is to establish a reversible mapping from the real text distribution to an unknown distribution. This unknown distribution could be set following a Gaussian distribution, avoiding problems like text discreteness and non-differentiability. Given the powerful performance of the AAE model, it could also be helpful for future research to convert other small-scale datasets to large-scale that are needed for improving the performance of machine learning models.

While AAE serves as an effective and commonly employed data augmentation strategy for time series, which is in the context of plant electrical signals, it has several constraints which may cause limitation of this work. First, AAE, like any generative model, can be prone to overfitting especially when the training data is limited. It makes the model under the risk of missing to generalizing well to new or unseen data. Second, interpreting the latent space of AAE can be challenging. It might be unclear what specific features are encoded in the latent variables.

## Conclusions

To summarize, using non-invasive electrophysiology, we generated three sets of wound-induced local and systemic electrical signals from plants treated with three light conditions (Normal, SED and LED). To develop new methods that could classify electrical signals more efficiently and precisely, we extracted different features from our generated signals and classified them using different machine learning models. We established the integral and deriv_1st as useful features for leaf 8 and leaf 13 SWPs classification under the three light conditions. In cooperation with our new proposed AAE method to augment the limited number of SWP samples, the Random Forest classifier showed the best performance in distinguishing both leaf 8 and leaf 13 SWPs with an accuracy of 0.99. In nature, plants are under constant mechanical stresses and their access to light is affected by various factors. Therefore, our model could also be a useful tool for crop plants in predicting their wound responses, even more broadly, other stress responses reflected by plant electrical signals.

### Supplementary Information

**Additional file 1: Fig S1.** The first-order derivative (deriv_1st) (A) and the integral (B) features of an example trace from a preprocessed leaf 8 (wounded) SWP. **Fig S2.** Network structures of the Q Encoder, P Decoder and D Discriminator. The circles filled with blue color represent neurons. **Fig S3.** Confusion matrices for prediction results using SVM classifier. **Fig S4.** Confusion matrices for prediction results using Random Forest classifier. **Table S1.** Time-domain features used in this study. **Table S2.** Triple classification results for leaf 8 (wounded) SWPs. **Table S3.** Triple classification results for leaf 13 (systemic) SWPs. **Table S4.** Classificaiton results for leaf 8 (wounded) SWPs upon data augmentation. **Table S5.** Classificaiton results for leaf 13 (systemic) SWPs upon data augmentation.

## Data Availability

The datasets used and/or analyzed during the current study are available from the corresponding author on reasonable request.
