# Supplementary material for: Early detection of dark-affected plant mechanical responses using enhanced electrical signals
Source: Plant Methods. 2024 Mar 26;20:49. doi: 10.1186/s13007-024-01169-4 (PMC10964643; doi:10.1186/s13007-024-01169-4)
Supplement: Supplementary file 1 — Additional file 1: Fig S1. The first-order derivative (deriv_1st) (A) and the integral (B) features of an example trace from a preprocessed leaf 8 (wounded) SWP. Fig S2. Network structures of the Q Encoder, P Decoder and D Discriminator. The circles filled with blue color represent neurons. Fig S3. Confusion matrices for prediction results using SVM classifier. Fig S4. Confusion matrices for prediction results using Random Forest classifier. Table S1. Time-domain features used in this study. Table S2. Triple classification results for leaf 8 (wounded) SWPs. Table S3. Triple classification results for leaf 13 (systemic) SWPs. Table S4. Classificaiton results for leaf 8 (wounded) SWPs upon data augmentation. Table S5. Classificaiton results for leaf 13 (systemic) SWPs upon data augmentation. [file 13007_2024_1169_MOESM1_ESM.docx]

**Supplementary Information for**

Early detection of dark-affected plant mechanical responses using enhanced electrical signals

Hongping Li, Nikou Fotouhi, Fan Liu, Hongchao Ji, Qian Wu


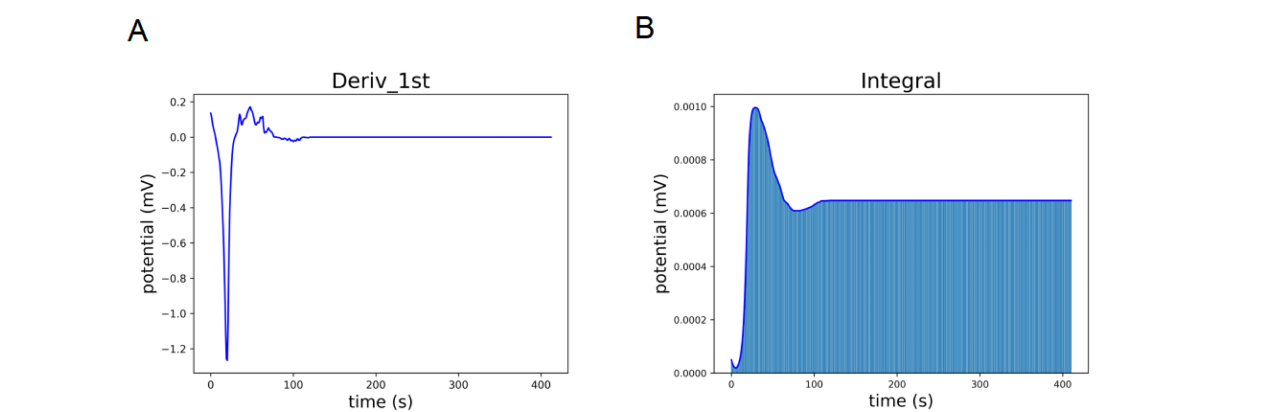


**Fig. S1** The first-order derivative (deriv_1st) (**A**) and the integral (**B**) features of an example trace from a preprocessed leaf 8 (wounded) SWP.


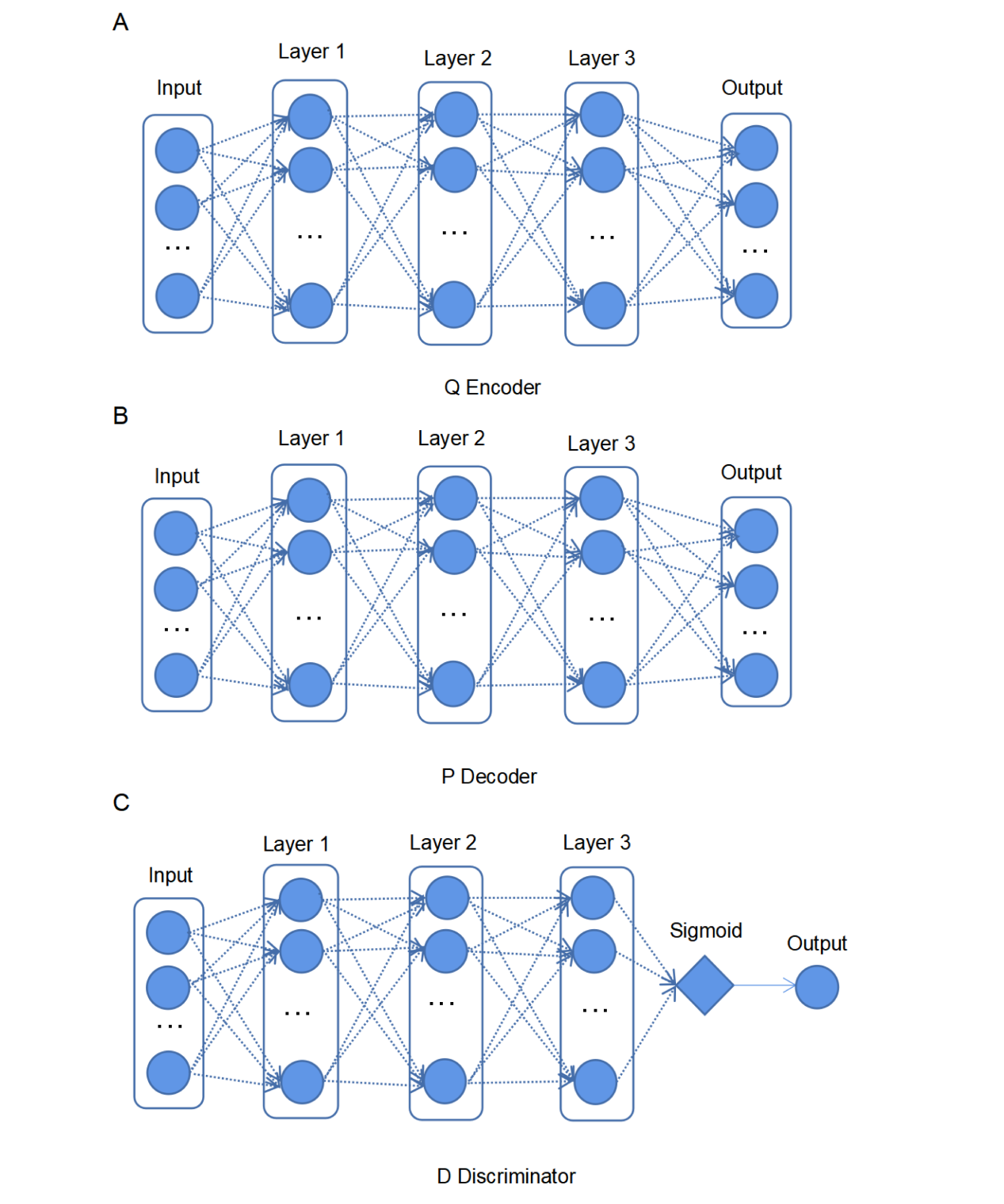


**Fig. S2** Network structures of the Q Encoder, P Decoder and D Discriminator. The circles filled with blue color represent neurons. **A** The Encoder consists of five linear layers. Each layer contains 411, 200, 100, 100, and 50 neurons respectively. **B** The Decoder consists of five linear layers. Each layer contains 50, 100, 100, 200, and 411 neurons respectively. **C** The Discriminator consists of five linear layers. Each layer contains 50, 200, 200, 200, and 1 neuron respectively.


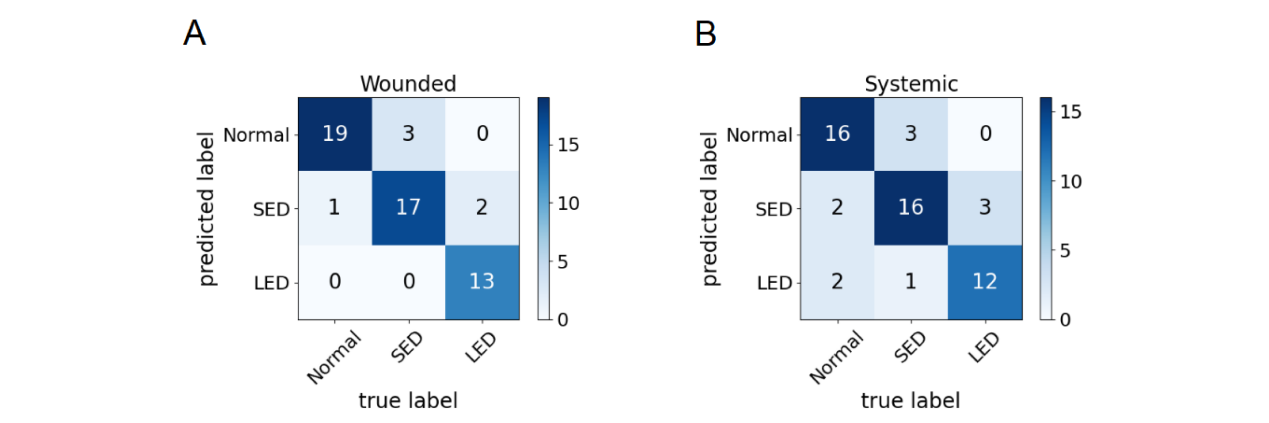


**Fig. S3** Confusion matrices for prediction results using SVM classifier**. A** Leaf 8 (wounded) SWPs. **B** Leaf 13 (systemic) SWPs. The color bars represent the percentage.


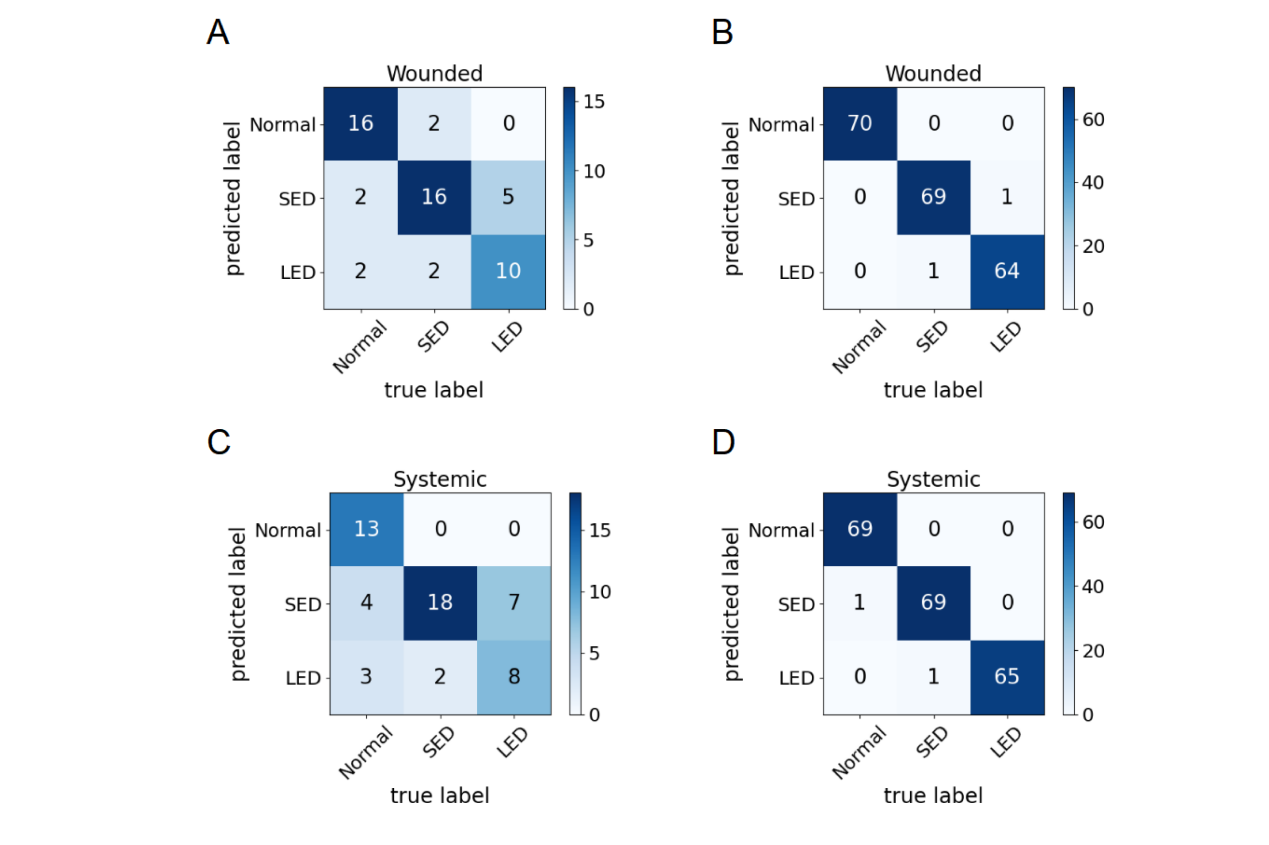


**Fig. S4** Confusion matrices for prediction results using Random Forest classifier. **A** Original leaf 8 (wounded) SWPs. **B** Extended leaf 8 (wounded) SWPs. **C** Original leaf 13 (systemic) SWPs. **D** Extended leaf 13 (systemic) SWPs. The color bars represent the percentage.

**Table S1** Time-domain features used in this study.

| **Features** |  |
| --- | --- |
| F1: Maximum | F2: Minimum |
| F3: Mean | F4: Variance |
| F5: Standard deviation | F6: Kurtosis |
| F7: Mean square root | F8: Area |
| F9: Decline_slope | F10: Rising_slope |
| F11: Amplitude | F12: Skewness |

**Table S2** Triple classification results for leaf 8 (wounded) SWPs. The best results are underlined and highlighted in bold.

| **Leaves** | **Features** | **Classifiers** | **Accuracy** | **Recall** | **Precision** | **F1 score** |
| --- | --- | --- | --- | --- | --- | --- |
| Leaf 8 | Time-domain | SVM | 0.72 | 0.73 | 0.73 | 0.73 |
|  |  | KNN | 0.71 | 0.71 | 0.71 | 0.71 |
|  |  | Random Forest | 0.63 | 0.64 | 0.67 | 0.65 |
|  |  | MLP | 0.58 | 0.60 | 0.58 | 0.57 |
|  | Deriv_1st | SVM | 0.69 | 0.68 | 0.68 | 0.68 |
|  |  | KNN | 0.58 | 0.59 | 0.57 | 0.57 |
|  |  | Random Forest | 0.76 | 0.76 | 0.77 | 0.76 |
|  |  | MLP | 0.62 | 0.63 | 0.63 | 0.61 |
|  | **Integral** | **SVM** | **0.89** | **0.89** | **0.90** | **0.89** |
|  |  | KNN | 0.56 | 0.53 | 0.54 | 0.49 |
|  |  | Random Forest | 0.56 | 0.55 | 0.61 | 0.56 |
|  |  | MLP | 0.71 | 0.71 | 0.72 | 0.71 |

**Table S3** Triple classification results for leaf 13 (systemic) SWPs. The best results are underlined and highlighted in bold.

| **Leaves** | **Features** | **Classifiers** | **Accuracy** | **Recall** | **Precision** | **F1 score** |
| --- | --- | --- | --- | --- | --- | --- |
| Leaf 13 | Time-domain | SVM | 0.60 | 0.58 | 0.58 | 0.58 |
|  |  | KNN | 0.60 | 0.59 | 0.59 | 0.59 |
|  |  | Random Forest | 0.71 | 0.71 | 0.71 | 0.71 |
|  |  | MLP | 0.65 | 0.63 | 0.63 | 0.63 |
|  | Deriv_1st | SVM | 0.67 | 0.65 | 0.66 | 0.65 |
|  |  | KNN | 0.51 | 0.47 | 0.46 | 0.43 |
|  |  | Random Forest | 0.71 | 0.69 | 0.75 | 0.70 |
|  |  | MLP | 0.55 | 0.53 | 0.52 | 0.52 |
|  | **Integral** | **SVM** | **0.80** | **0.80** | **0.80** | **0.80** |
|  |  | KNN | 0.56 | 0.57 | 0.61 | 0.57 |
|  |  | Random Forest | 0.69 | 0.69 | 0.69 | 0.69 |
|  |  | MLP | 0.65 | 0.65 | 0.65 | 0.65 |

**Table S4** Classificaiton results for leaf 8 (wounded) SWPs upon data augmentation. The results before augmentation are shown before slash. The best results are underlined and highlighted in bold.

| **Leaves** | **Features** | **Classifiers** | **Accuracy** | **Recall** | **Precision** | **F1 score** |
| --- | --- | --- | --- | --- | --- | --- |
| Leaf 8 | Time-domain | SVM | 0.72/0.82 | 0.73/0.82 | 0.73/0.83 | 0.73/0.81 |
|  |  | KNN | 0.71/0.81 | 0.71/0.81 | 0.71/0.82 | 0.71/0.81 |
|  |  | Random Forest | 0.63/0.80 | 0.64/0.81 | 0.67/0.81 | 0.65/0.80 |
|  |  | MLP | 0.58/0.60 | 0.60/0.59 | 0.58/0.60 | 0.57/0.60 |
|  | **Deriv_1st** | SVM | 0.69/0.96 | 0.68/0.96 | 0.68/0.95 | 0.68/0.95 |
|  |  | KNN | 0.58/0.90 | 0.59/0.90 | 0.57/0.90 | 0.57/0.90 |
|  |  | **Random Forest** | **0.76/0.99** | **0.76/0.99** | **0.77/0.99** | **0.76/0.99** |
|  |  | MLP | 0.62/0.90 | 0.63/0.90 | 0.63/0.90 | 0.61/0.90 |
|  | Integral | SVM | 0.89/0.96 | 0.89/0.96 | 0.90/0.95 | 0.89/0.95 |
|  |  | KNN | 0.56/0.85 | 0.53/0.85 | 0.54/0.86 | 0.49/0.85 |
|  |  | Random Forest | 0.56/0.91 | 0.55/0.91 | 0.61/0.91 | 0.56/0.91 |
|  |  | MLP | 0.71/0.90 | 0.71/0.90 | 0.72/0.90 | 0.71/0.90 |

**Table S5** Classificaiton results for leaf 13 (systemic) SWPs upon data augmentation. The results before augmentation are shown before slash. The best results are underlined and highlighted in bold.

| **Leaves** | **Features** | **Classifiers** | **Accuracy** | **Recall** | **Precision** | **F1 score** |
| --- | --- | --- | --- | --- | --- | --- |
| Leaf 13 | Time-domain | SVM | 0.60/0.88 | 0.58/0.88 | 0.58/0.88 | 0.58/0.88 |
|  |  | KNN | 0.60/0.89 | 0.59/0.89 | 0.59/0.89 | 0.59/0.89 |
|  |  | Random Forest | 0.71/0.89 | 0.71/0.89 | 0.71/0.89 | 0.71/0.89 |
|  |  | MLP | 0.65/0.76 | 0.63/0.75 | 0.63/0.75 | 0.63/0.73 |
|  | **Deriv_1st** | SVM | 0.67/0.94 | 0.65/0.94 | 0.66/0.94 | 0.65/0.94 |
|  |  | KNN | 0.51/0.87 | 0.47/0.87 | 0.46/0.88 | 0.43/0.87 |
|  |  | **Random Forest** | **0.71/0.99** | **0.69/0.99** | **0.75/0.99** | **0.70/0.99** |
|  |  | MLP | 0.55/0.93 | 0.53/0.93 | 0.52/0.93 | 0.52/0.93 |
|  | Integral | SVM | 0.80/0.97 | 0.80/0.97 | 0.80/0.97 | 0.80/0.97 |
|  |  | KNN | 0.56/0.91 | 0.57/0.91 | 0.61/0.91 | 0.57/0.91 |
|  |  | Random Forest | 0.69/0.96 | 0.69/0.96 | 0.69/0.96 | 0.69/0.96 |
|  |  | MLP | 0.65/0.95 | 0.65/0.95 | 0.65/0.95 | 0.65/0.95 |
